# The Unemployed

**Published:** 1888-09-01

**Authors:** John Law

**Affiliations:** 9, Ludgate Square, E.C.


### THE UNEMPLOYED.

Sir,—I have just seen the number of The Hospital dealing with the question of the unemployed; but I gather you approve of individual effort more than legislation. My opinion is, that we want men in the House to draw attention to labour questions, and I am doing all I can in that direction. For the last year I have lived among the unemployed,—hence I have not seen your paper. But directly I read your notice of "Out of Work," sent to me by the publisher, I felt you must have the interest of these unfortunate people at heart; and your articles prove you to be wise, as well as kind-hearted. Accept my hearty thanks. John Law.

9, Ludgate Square, E.C., Aug. 25, 1888.

